# Assessment of Metalloid and Metal Contamination in Soils from Hainan, China

**DOI:** 10.3390/ijerph15030454

**Published:** 2018-03-03

**Authors:** Xiangjun Liao, Chao Zhang, Guangyi Sun, Zhonggen Li, Lihai Shang, Yangrong Fu, Yusheng He, Yi Yang

**Affiliations:** 1 Geological Bureau of Hainan Province, Haikou 570206, China; xiangjun_liao@163.com; 2 State Key Laboratory of Environmental Geochemistry, Institute of Geochemistry Chinese Academy of Sciences, Guiyang 550002, China; zhangchao@mail.gyig.ac.cn (C.Z.); lizhonggen@mail.gyig.ac.cn (Z.L.); shanglihai@mail.gyig.ac.cn (L.S.); 3 University of Chinese Academy of Sciences, Beijing 100049, China; 4 Hainan Institute of Geological Survey, Haikou 570206, China; fuyangrong2013@163.com (Y.F.); heyusheng2013@163.com (Y.H.); yangyi2013hk@163.com (Y.Y.)

**Keywords:** metals, arsenic, soils, Hainan, source

## Abstract

The characterization of the concentrations and sources of metals and metalloids in soils is necessary to establish quality standards on a regional level and to assess the potential threat of metals to food safety and human health. A total of 8713 soil samples throughout Hainan Island, China were collected at a density of one sample per 4 km^2^, and concentrations of As, Cd, Cr, Cu, Hg, Ni, Pb, Se, and Zn were analyzed. The geometric mean values of the elements were 2.17, 0.60, 26.5, 9.43, 0.033, 8.74, 22.2, 0.26, and 39.6 mg·· kg^−1^ for As, Cd, Cr, Cu, Hg, Ni, Pb, Se, and Zn, respectively, significantly lower than the background values of Chinese soils with the exception of Se. Principal component analysis (PCA) suggested that multiple anthropogenic sources regulated the elemental compositions of the Hainan environment. Coal combustion and mining are important anthropogenic sources of metals for Hainan. The geochemical maps of elements in Hainan soils were produced using the Geographic Information System (GIS) method, and several hot-spot areas were identified. The ecological impact of As, Cd, Cu, Cr, Hg, Pb, Ni, and Zn pollution to the soils was extremely “low”.

## 1. Introduction

With the rapid industrial development, population expansion, and insufficiency of pollution control, soil trace metal contamination is increasingly becoming a global problem at private as well as governmental levels, especially as soils constitute a crucial component of rural and urban environments [1,2] which can be considered as an important “ecological crossroad” in the landscape [3,4]. Trace metals in soils and dusts can be accumulated in the human body via direct inhalation, ingestion, and dermal contact absorption [5,6,7,8] or via the soil-crop system [9,10]. The anthropogenic sources of metals include traffic emission, industrial and domestic emission, atmospheric deposition, mining, waste disposal, sewage, pesticides, and fertilizers [11,12,13,14]. Without proper management, abandoned mines will cause more serious environmental impacts compared with active mines [15]. Consumption of food crops growing in contaminated area is one of the important sources of human exposure to metals in mining areas [16,17,18].

Depending on the different counties and different geological backgrounds, the regulations on soil metal contents at the national and international levels are quite different. Compared with the background values of Chinese soils [19], the class I value of the Environmental Quality Standard for Soils in China [20] were quite rigorous when compared with other international regulations on soil metal, such as the Values for soil remediation proposed by Dutch Ministry of Housing [21] and Canadian Soil Quality Guidelines for the Protection of Environmental and Human Health [22].

Hainan Island, located in the southernmost part of China, is one of the least polluted regions in the country [23]. However, in recent decades, this once-clean region has received notable anthropogenic pollutant inputs, especially metal/metalloids pollutants. On Hainan Island, there are three types of ecosystems including urban, agricultural, and mining areas. In our study, metals and metalloids in the soils of different ecosystems from Hainan Island were studied for the purpose of monitoring the impact of human activities on the ecosystems.

## 2. Materials and Methods

### 2.1. Study Area

**Figure 1:**
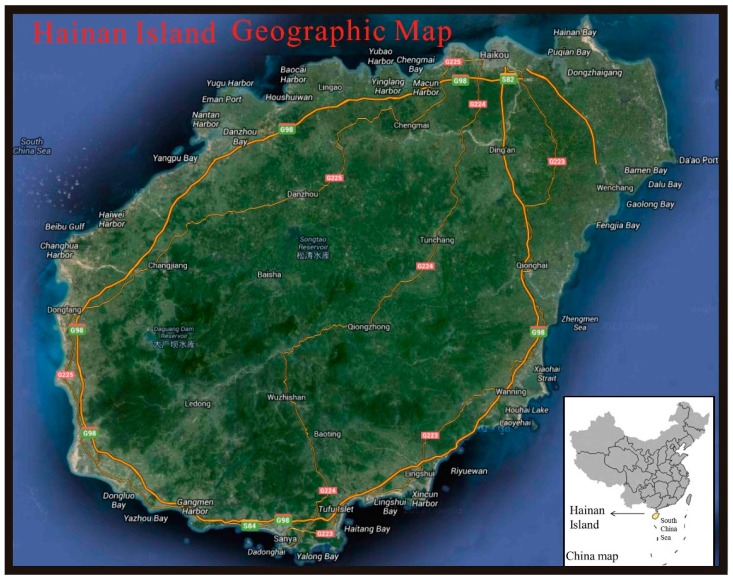

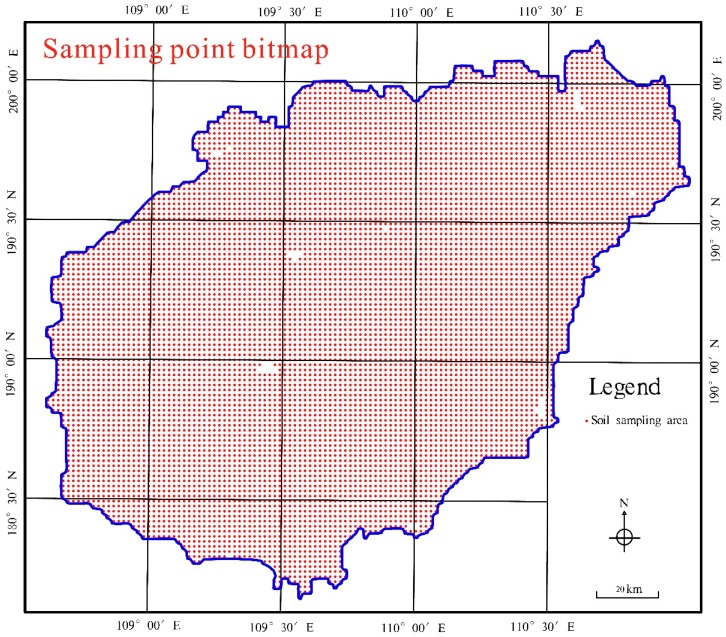
Location of the sampling sites.

The study area (Figure 1), Hainan Island, is located in southern China (18°10′–20°10′ N, 108°37′–111°03′ E) and covers 33,920 km^2^ of land area. The terrain in the east is higher than that of the west. The step-like landforms are prominent with a succession of littoral plain, terrace, platform, hill, low mountain, and middle mountain from east to west. The central mountain of the Five Finger Mountain is the highest one on the island with an altitude of 1867 m above sea level. The island has a semi-arid tropical monsoon climate, which has obvious dry seasons and wet seasons. The climate in this area is tropical monsoon with an average annual rainfall of 1600–2500 mm and average annual temperatures of 23–25 °C [24].

### 2.2. Sample Collection and Analysis

A total of 8713 soil samples (0–20 cm) (Figure 1) were collected from Hainan Island. Sampling was based on the specifications of the National Multi-Purpose Regional Geochemical Survey carried out by China Geological Survey (CGS) in 2005. About 1 kg was collected from a 4 × 4 km grid, and each sample consisted of about four subsamples from the sample plot. Samples were collected using a stainless-steel hand auger, which was plated with a Teflon coating. The coordinates of the sample locations were recorded with a portable Global Positioning System (GPS), and the sampling sites are shown in Figure 1. The soil types of our 8713 samples included coastal sandy soil, coastal saline soil, latosolic red soil, yellow soil, volcanic ash soil, chisley soil, paddy soil, alluvial soil, dry red soil, latosol, and purple soil. The mean pH value of the study area was 5.52 ± 0.74, with maximum and minimum values of 9.10 and 3.47, respectively.

All samples were air-dried at room temperature (20–25 °C) and then sieved to 2 mm to remove large plant roots and gravel-sized materials before analysis. Concerning chemical analysis of Hg and As, 0.1–0.3 g of each sample were digested at 95 °C in a water bath for 3 h using 5 mL aquaregia (a mixture of HCl and HNO_3_ at 3:1 *v*/*v*). An appropriate aliquot of the digest was analyzed using cold vapor atomic fluorescence spectrophotometry (CVAFS; for Hg, Tekran Model 2500, Tekran Instruments Corp., Knoxville, TN, USA; for As, AFS-920, Beijing Jitian Instrument Corp., Beijing, China).

For Se digestion, subsamples (approximately 0.05 g) were accurately weighed into Teflon tubes. Concentrated nitric acid (3 mL of sub-distilled, 16 mol·L^−1^) and concentrated hydrofluoric acid (0.5 mL of sub-distilled, 23 mol·L^−1^) were added to each tube. After 3 h, the closed tubes were heated for 8 h at 155 °C in an oven. After cooling, 2 mL of H_2_O_2_ (30%) were added and the solution recapped and heated for 45 min at 90 °C. Each digest solution was then transferred into a 15 mL Polyfluoroalkoxy (PFA) container and evaporated on a hot plate at 90 °C to near dryness. Selenium in each digest solution was transformed into Se (IV) by adding 2.4 mL of 5 mol·L^−1^ HCl (ultrapure grade) followed by incubation at 95–100 °C for 45 min. Each solution was finally diluted to 16 mL with ultrapure water (Milli-Q Reference Ultra Water Purification System, MILLIPORE, France). The total Se concentration of the plant and soil digest solutions was quantified using an atomic fluorescence spectrometer (AFS) equipped with a sequential injection hydride generation (HG) sampling system (AFS-920, Beijing Jitian Instrument Corporation, Beijing, China).

For the analysis of remaining trace metal/metalloids, a wet digestion procedure was used coupled with inductively coupled plasma-mass spectrometry (ICP-MS, ELAN DRC-e, PerkinElmer Inc., Montreal, QC, Canada). Briefly, 50 mg of sample were digested using 1 mL of HF and 1 mL of HNO_3_ in the Poly tetra fluoroethylene (PTFE)-lined stainless-steel bombs heated to 190 °C for 24 h. Insoluble residues were dissolved using 6 mL of 40% *v*/*v* HNO_3_ heated to 140 °C for 5 h. After cooling down, about 0.4 mL of the digest was transferred to a centrifuge tube, and 500 ng rhodium in liquid solution was added. Then, Milli-Q water was added to reach a total volume of approximately 10 mL. Rhodium was used as an internal standard to correct for matrix effects and instrumental drift. All the reagents used were trace metal grade.

Quality assurance and quality control were determined by method blanks, duplicates, and certified reference materials (CRM) (sediment and soil CRM: GBW07305, GBW07405, and NIST 2710). Recovery from CRM sediment (GBW07305) was between 99% and 102%, from CRM soil (GBW07405) between 92% and 107% and from CRM soil (NIST 2710) between 95% and 106%. The relative percentage difference was less than 8% for duplicate samples. The Hg concentration in solution was determined using the dual-stage gold amalgamation method and cold vapor atomic fluorescence spectrometry (CVAFS) detection. The limit of detection (LOD) was 0.013 ng·g^−1^. The total Se concentration of the plant and soil digest solutions was quantified using an atomic fluorescence spectrometer (AFS) equipped with a sequential injection hydride generation (HG) sampling system (Beijing Titan Instrumentals Co. Ltd., Beijing, China). The limit of detection (LOD) was 0.2 μg·L^−1^. The other trace metal/metalloids were analyzed by a wet digestion procedure coupled with inductively coupled plasma-mass spectrometry (ICP-MS, ELAN DRC-e, PerkinElmer Inc., Montreal, QC, Canada). The limits of detection of the different elements were lower than 1 μg·L^−1^.

### 2.3. Statistical Analysis

The statistical analysis of data was performed using the statistical package Statistical Product and Service Solutions (SPSS) 15.0 for Windows (SPSS Inc., New York, NY, USA). The methods included one-way analysis of variance (ANOVA), Mann-Whitney test, and principal component analysis (PCA). Statistical significance was determined at alpha = 0.05. Nonparametric tests were conducted to determine the differences. A geochemical map was developed using GIS (MapGis 6.7, Zondy Cyber Group Co., Ltd., Wuhan, China).

### 2.4. Potential Ecological Risk Assessment

The method of potential ecological risk assessment was first introduced by Håkanson [25,26] and has been widely applied to pollution assessment of sediment and soil [27]. Håkanson’s method is based on the hypothesis that an ecological risk index for toxic substances should account for the following requirements: (1) the potential ecological risk index (RI) increases with an increase in metal(loid) pollution in the substrate; (2) the potential for causing ecological harm of different metal(loid)s in the substrate have synergistic effects, and the potential risk of the resultant synergistic damage shall be more serious; and (3) the toxicity response of each element varies, and those metal(loid)s exerting greater toxicity shall play a larger role in the RI. Hence, the process integrates concentration and ecological toxicity of different pollutants and then gives a potential ecological risk index for the study area, as shown in

$$RI=\sum_{i=1}^{n}E_{i}=\sum_{i=1}^{n}T_{i}\times C_{i}$$

where *RI* is the potential ecological risk index for the study area; *E_i_* is the potential ecological risk factor for a given pollutant (i); *T_i_* is the “toxic-response” factor for a given pollutant as calculated by Håkanson [26] and Xu et al. [28], i.e., Hg = 40, Cd = 30, As = 10, Pb = Cu = Ni = 5, Cr = 2, Zn = 1; and *C_i_* is the ratio of metal(loid) concentration in the soil to the corresponding background in Chinese soils. The RI index is divided into four levels according to Håkanson’s [26] method, namely, *RI* < 150, low ecological risk for the study area; 150 ≤ *RI* < 300, moderate ecological risk for the study area; 300 ≤ *RI* < 600, considerable ecological risk for the study area; *RI* > 600, very high ecological risk for the study area.

## 3. Results and Discussion

### 3.1. Concentrations of Metal(loid)s

The concentrations of metal(loid)s in the soils in Hainan and their descriptive statistical results are listed in Table 1. The results of the P-P test showed that As, Cd, Cr, Cu, Hg, Ni, Pb, Se, and Zn concentrations fit a lognormal distribution. The geometric mean values were adopted for analyses. The metal(loid)s showed a wide variety of concentrations. The geometric mean concentrations of As, Cd, Cr, Cu, Hg, Ni, Pb, Se, and Zn were 2.17, 0.06, 26.5, 9.43, 0.033, 8.74, 22.2, 0.26, and 39.6 mg kg^−1^, respectively, which were significantly lower than the background values of Chinese soils [19,29], except for Se. Compared with the values for soil remediation proposed by the Dutch Ministry of Housing, Spatial Planning and Environment [21] and Canadian Soil Quality Guidelines for the Protection of Environmental and Human Health for Residential [22], the geometric mean contents of all the seven elements were much lower than the two guidelines. However, it should be noted that the concentrations of metals in substantial soil samples (As 6.4%, Cd 4.9%, Cr 15.6%, Cu 13.0%, Hg 1.3%, Ni 14.1%, Pb 21.0%, Zn 10.8%) of Hainan are higher than the first grade of the soil standard of China [20]. The anthropogenic inputs might be responsible for the presence of these elements in soils.

The geometric means of the elements of various soil types of Hainan Island are given in Table 2 and indicate that the means of most of the nine trace metal/metalloids in coastal saline soil are the lowest, except for Cr, Hg, and Ni. The means of most elements in dry red soil are the next lowest; while volcanic ash soil has the highest mean values for most of elements, except for As, Cd, Hg, Pb, and Se. The chisley soil has the second highest mean values for most elements. The highest As geometric mean was observed in yellow soil, and the highest Pb geometric mean was found in latosolic red soil. In general, the sequence of elemental mean values by soil order is: volcanic ash soil > chisley soil > yellow soil ≈ latosolic red soil > latosol > paddy soil > alluvial soils > purple soil > coastal sandy soil < dry red soil < coastal saline soil.

**Figure 2:**
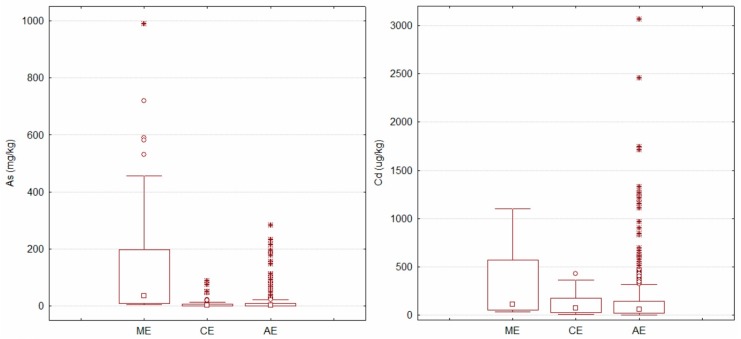

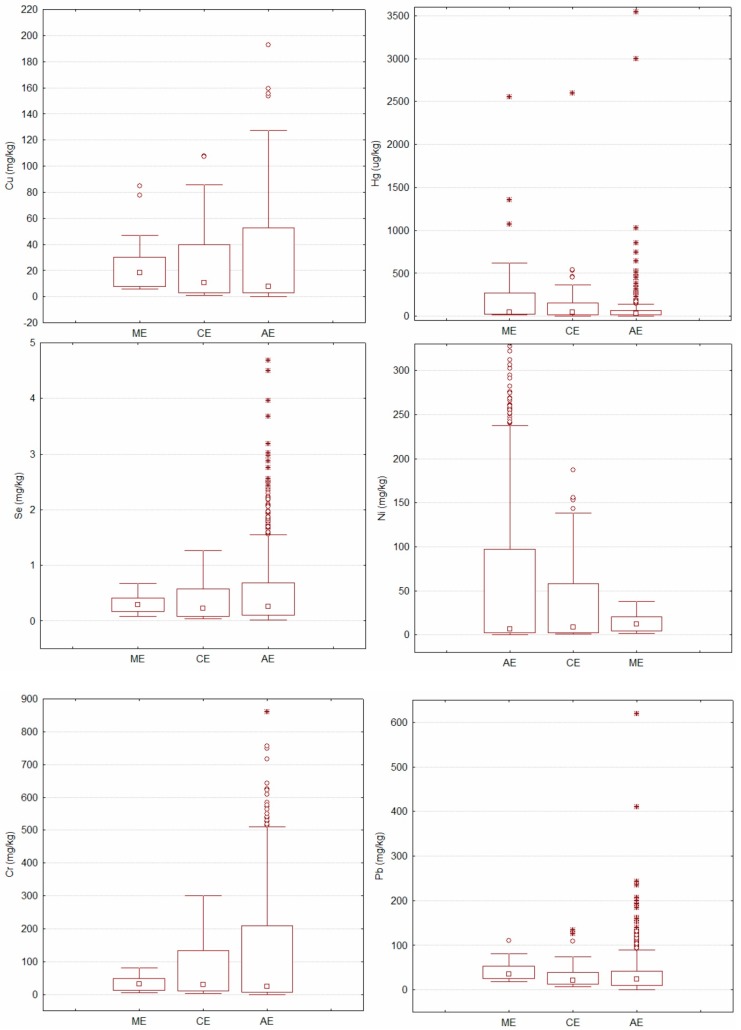

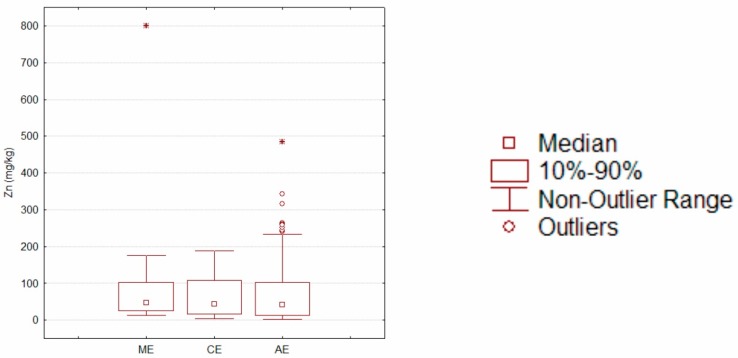
Box-and whisker plots of As, Cd, Cu, Cr, Hg, Pb, Ni, Se, and Zn in soils under different patterns of ecosystem. ME = mining ecosystem; CE = city ecosystem; AE = agricultural ecosystem.

Based on the main human activities and different land usage functions, three types of ecosystems were selected in this study: agricultural ecosystem (AE), mining ecosystem (ME), and city ecosystem (CE). As shown in Figure 2, the mean concentrations of metal(loid)s in ME decreased in the order of As > Zn > Pb > Cr > Cu > Ni > Se > Cd > Hg, which were 95.05, 66.24, 38.85, 29.80, 20.11, 12.20, 0.29, 0.23, and 0.13 mg·kg^−1^, respectively. Two independent sample tests (Mann-Whitney *U*) showed that the concentrations of As, Cd, Hg, Pb, and Zn in ME were significantly higher than those in the other two types of ecosystem (*p* < 0.05). For the other two types of ecosystems, tests on several independent samples (Kruskal-Wallis *H*) were conducted, and the result indicated that only the concentrations of Hg showed significant differences between AE and CE. The mean value of Hg in CE (0.085 mg·kg^−1^) was significantly higher than that in AE (0.041 mg·kg^−1^), indicating an obvious accumulation of Hg in CE.

### 3.2. Source Identification

From the descriptive statistical analysis, the elevated concentrations of As, Cd, Hg, Pb, and Zn in ME and Hg in CE might indicate anthropogenic sources of these elements. However, the trace metals in agricultural ecosystems were lower than or approximately equal to the background values of Chinese soils, suggesting less influence from anthropogenic activities (a natural origin). Principal component analysis (PCA) is a useful method to identify anthropogenic pollution sources in different ecosystem settings (Table 3, Ba, Co, Fe, S, Sb, and V are not published date).

For CE, three factors, with eigen values > 1, have a cumulative variance of more than 73%. Principal component (PC3) exhibits high loading for Cd and S with a maximum contribution of 13.67% of total variance. In addition, Hg has a higher loading (0.366) in these three factors. Mercury, Cd, and S in PC3 may be an indicator of the coal combustion source since the coal burning emission was regarded as an important source of Hg and S in an urban environment [30,31]. The coal combustion accounted for 33.4% of all energy consumption in 2011 in Hainan [32]. PC1, dominated by Co, Cr, Cu, Fe, Ni, V, and Zn, accounts for 37.4% of the total variance. PC2 is dominated by As, Ba, and Pb, accounting for 22.12% of the total variance. Compared with background values of Chinese soils, As, Cd, Cr, Cu, Ni, Pb, Se, and Zn have concentrations approximate to their corresponding background values, while mercury has extremely elevated concentrations in CE. So, PC1 and PC2 indicate a natural origin.

For ME, three factors are obtained with eigenvalues >1, cumulatively contributing to 78.01% of total variance. The overwhelming 39.05% of the total variance is contributed by PC1, showing higher loadings for Co, Cr, Cu, Fe, Ni, Se, and V. These metals in PC1 may indicate the influence of iron/steel smelting and processing activities because they are the principal industries in the studied area [32,33]. PC2 contributes As, Hg, Pb, S, and Sb at 27.45% total variance. The association of the trace metals of Pb, Hg, S, Sb, and As in PC2 is likely related to the influence of coal combustion in smelting production or other activities [31,34,35,36]. The PC3, with a variance of 11.51%, represented by Zn, Cd, and Ba, may be an indicator of traffic emission sources. Ba was normally regarded as a marker for automobile exhaust [37]. Zn can originate from the wear and tear of vulcanized vehicle tires and corrosion of galvanized automobile parts [38].

For AE, concentrations of the metals in most samples are slightly higher than, or close to, their corresponding background values in Chinese soils. This suggests that these metals in Hainan soils mainly originated from nature.

Overall, PCA suggested that multiple anthropogenic sources regulated the elemental compositions of the Hainan environment [39].

### 3.3. Spatial Distributions and Risk Assessment

**Figure 3:**
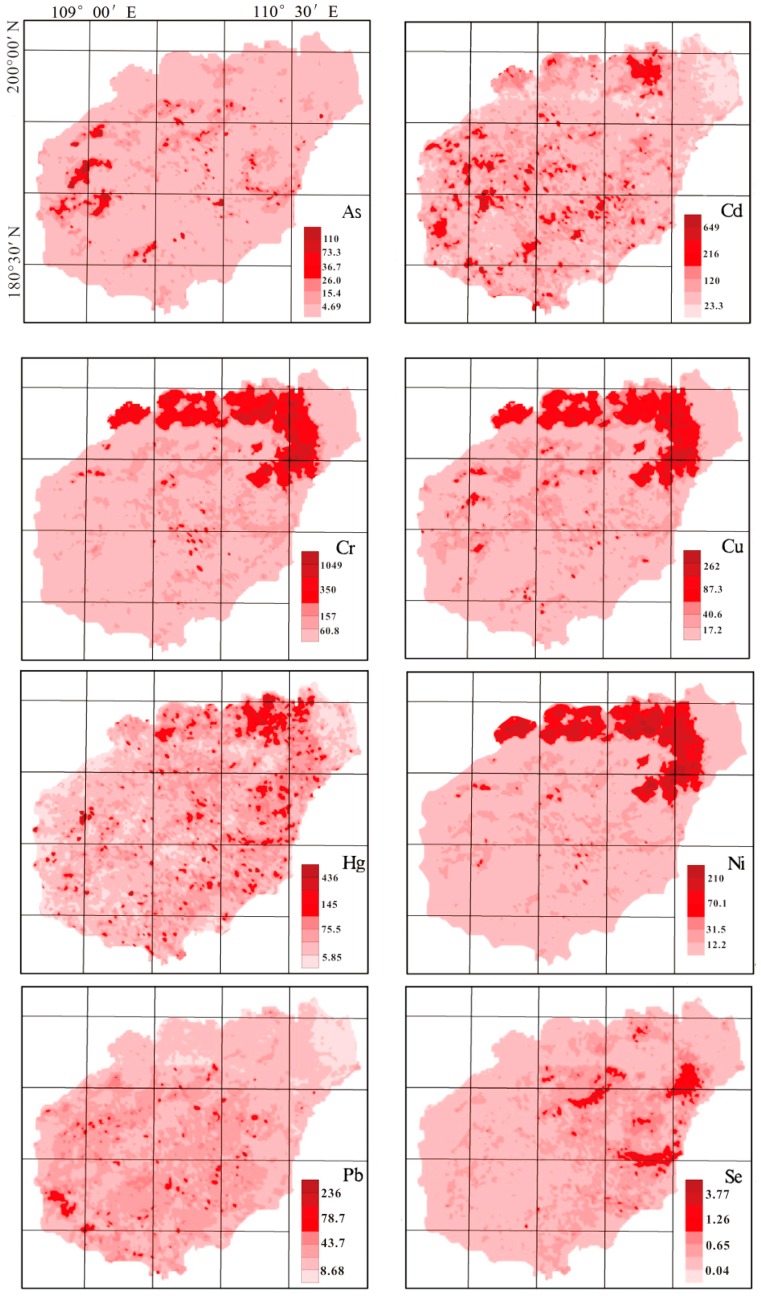

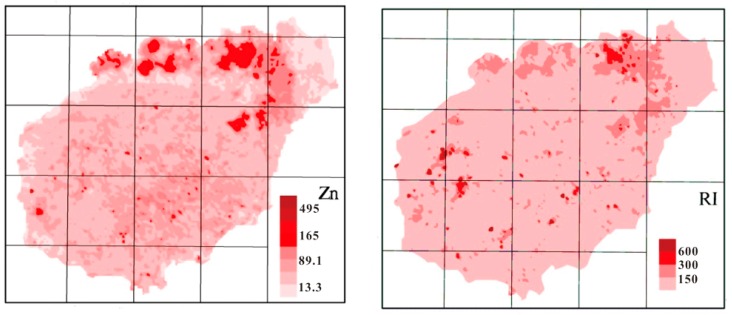
Spatial distribution of As, Cu, Cr, Pb, Ni, Se, Zn (mg kg^−1^), Cd, and Hg (μg kg^−1^) and risk index (RI) in soils of Hainan Island.

The soil pollution maps of metals including As, Cd, Cu, Cr, Hg, Pb, Ni, Zn, and Se were generated using GIS [40,41,42]. The results of the elements are shown in Figure 3. The spatial distributions of metals such as Cr, Cu, Ni, and Zn in soils were similar in Hainan soils with high concentrations in the northeast area of Hainan (a hook shaped distribution). However, Cu was not in the same factor in the statistical PCA results. Copper, Cr, Hg, and Zn may originate from different sources and most probably from anthropogenic inputs. Moreover, the spatial distribution of As and Cd (see Figure 3) was distinctly different from that of the trace elements such as Cu, Cr, Hg, Pb, Zn, and Se. High concentrations of As and Cd were mainly found to be dispersed. Furthermore, the spatial distribution of Pb appeared to have a few hotspots of high concentrations but generally had low estimated metal concentrations throughout the majority of the Hainan area, while the distribution of Se had more areas of high metal concentrations that were located near the high values of Cr, Cu, Hg, and Zn. Therefore, as discussed above, As, Cd, Pb, and Se may be attributed to the inputs from natural sources.

The study area was highly enriched with some elements, including As, Cd, Cu, Cr, Hg, Pb, Ni, and Zn. Potential ecological risk index (RI) was used to assess the degree of trace metal pollution in soils (Figure 3). In general, the range of RI spanned from 6.84 to 3635, with a mean value of 99.22 for the whole area. The relatively high pollution areas were mainly located in Changmai County (northeast) and Changjiang County (west). This may be related to the local mining and smelting (where the counties are located near the mining site). The ecological impact of As, Cd, Cu, Cr, Hg, Pb, Ni, and Zn pollution to the soils was overwhelmingly “low”, with 85.9% samples feathered with low ecological risk, 12.3% moderate ecological risk, 1.45% considerable ecological risk, and 0.36% very high ecological risk.

For different ecosystems, RI in ME is the highest (mean: 346.0), followed by CE (mean: 144.5), and in AE, RI is lowest (mean: 95.9), which can be regarded as a low ecological risk for agriculture. For ME, about 55.4% of soils samples were feathered with moderate or even high ecological risk. For CE, only 5.9% of soil samples were feathered with considerable ecological risk.

The contribution of different elements to the potential ecological risk index (RI) decreased in the following order: Hg (40.9) > Cd (32.0) > Pb (5.5) > Ni > As > Cu > Cr > Zn for AE; Hg (85.1) > Cd (36.6) > As (5.7) > Pb > Ni > Cu > Cr > Zn for CE; and Hg (130.1) > As (103.3) > Cd (94.6) > Pb (8.2) > Cu > Ni > Cr > Zn for ME. For the three ecosystems, mercury and Cd are the main pollutants impacting on human health. Furthermore, for ME, the pollution of As cannot be ignored.

## 4. Conclusions

The results obtained in this work offered relevant information on the distribution, sources, and extent of metal and metalloid element accumulation in soils of Hainan Island. The trace metal and metalloid concentrations were mostly within lognormal ranges.

Compared with the background values of Chinese soils, the geometric mean concentrations of trace metals were significantly lower, while the geometric mean value of Se was higher. In addition, it should be noted that the trace metal concentrations in soils of Hainan are generally higher (As 6.4%, Cd 4.9%, Cr 15.6%, Cu 13.0%, Hg 1.3%, Ni 14.1%, Pb 21.0%, Zn 10.8%,) than the first grade of the standard.

There is indeed serious pollution of As, Cd, Hg, Pb, and Zn in ME and a mild pollution problem for the other two types of ecosystem. Variation in the element concentrations in soils of Hainan Island have both natural and anthropogenic origins. PCA suggested that multiple anthropogenic sources regulated the elemental compositions of the Hainan environment. Coal combustion and mining are large anthropogenic sources for Hainan. The geochemical maps of elements in Hainan soils were produced using the GIS method, and several hot-spot areas were identified. The ecological impact of As, Cd, Cu, Cr, Hg, Pb, Ni, and Zn pollution to the soils was extraordinarily “low”.

In addition, the ecological and health implications of these findings should be considered in future investigations and provide a basis for effectively targeting policies to protect soils from long-term trace metal accumulation.

## Figures and Tables

**Table 1:** Statistics of studied elements (mg·kg^−1^) in soils (0–20 cm) of Hainan Island.

	As	Cd ^a^	Cr	Cu	Hg ^a^	Ni	Pb	Se	Zn
N	8713	8713	8713	8713	8713	8713	8713	8713	8713
Minimum	0.01	2	0.1	0.25	1	0.1	1	0.02	2
Maximum	988.04	3064	860.3	192.9	3540	327.1	619.6	4.68	800
Geometrical mean	2.17	60.19	26.50	9.43	33.34	8.74	22.20	0.26	39.64
Variation coefficient	4.05	1.16	1.58	1.35	1.77	1.91	0.67	0.88	0.74
Std. Deviation	21.42	93.61	96.32	23.34	75.20	48.18	17.51	0.30	37.91
Skewness	24.91	10.52	2.65	2.35	29.30	2.74	7.79	3.39	2.51
Kurtosis	858.68	219.08	7.09	4.97	1127.52	6.93	191.20	22.67	22.31
China BK ^b^	9.20	74.00	53.90	20.00	40.00	23.40	23.60	0.22	67.70
Threshold of the first grate ^c^	15	200	90	35	150	40	35		100
Canadian soil quality guidelines ^d^	12	10000	64	63	6600	45	140	1	200
Target value of Dutch soil guidelines ^e^	29	800	100	36	300	35	85		140

^a^ μg·kg^−1^; ^b^ Background values of Chinese soils [19], A layer (0–20 cm), more than 4000 samples; ^c^ Class I value of the Environmental Quality Standard for Soils in China [20]; ^d^ Canadian Soil Quality Guidelines for the Protection of Environmental and Human Health (Residential) [22]; ^e^ Values for soil remediation proposed by Dutch Ministry of Housing, Spatial Planning and Environment [21].

**Table 2:** Geometrical means of elements in soils (0–20 cm) of Hainan Island of different soil types (means are mg·kg^−1^).

Soil Types	Number of Samples	As	Cd ^a^	Cr	Cu	Hg ^a^	Ni	Pb	Se	Zn
Coastal sandy soil	311	2.69	44.76	21.72	5.87	29.22	7.08	14.20	0.11	18.61
Coastal saline soil	12	1.40	28.42	16.30	3.19	65.75	3.66	11.53	0.10	14.45
Latosolic red soil	890	5.14	110.62	24.16	9.48	39.00	8.15	34.26	0.31	58.85
Yellow soil	255	7.24	131.18	25.15	9.39	42.60	8.32	33.86	0.31	57.66
Volcanic ash soil	5	2.00	177.64	293.07	65.06	90.97	154.88	15.45	0.33	143.07
Chisley soil	50	3.00	223.30	240.41	52.13	96.64	128.77	16.62	0.47	129.30
Paddy soil	874	3.07	60.27	74.11	19.44	41.60	30.71	18.56	0.28	42.19
Alluvial soil	90	3.71	96.24	28.87	11.36	39.30	10.03	25.91	0.19	38.91
Dry red soil	250	1.96	73.70	12.68	5.30	24.43	3.40	26.27	0.12	23.00
Latosol	5726	6.01	74.35	62.63	18.02	43.01	25.30	26.90	0.39	51.12
Purple soil	76	3.50	79.51	32.50	12.77	29.45	8.93	21.21	0.28	34.96

^a^ means are μg·kg^−1^.

**Table 3:** Rotated factor loadings (varimax normalized) of selected metals in CE samples (*n* = 168) and ME samples (*n* = 83).

Components	CE Samples			ME Samples		
	PC1	PC2	PC3	PC1	PC2	PC3
As	0.036	0.853	−0.001	0.304	0.753	−0.096
Ba	−0.023	0.721	0.380	−0.293	0.165	−0.631
Cd	0.110	0.314	0.797	0.261	0.470	0.681
Co	0.936	−0.017	0.135	0.939	0.074	−0.074
Cr	0.966	−0.084	−0.049	0.875	0.297	0.240
Cu	0.752	0.513	0.212	0.802	0.329	0.248
Fe	0.964	0.177	0.041	0.938	0.125	0.131
Hg	0.003	0.097	0.366	0.047	0.861	0.086
Ni	0.961	−0.116	0.043	0.951	0.171	0.154
Pb	−0.142	0.707	0.427	0.057	0.867	0.116
S	0.030	−0.120	0.679	0.216	0.834	0.273
Sb	0.065	0.879	0.193	0.298	0.822	0.150
Se	0.214	0.536	−0.214	0.716	0.346	0.025
V	0.954	0.093	−0.077	0.939	0.016	0.205
Zn	0.627	0.254	0.579	−0.031	0.268	0.728
Eigenvalue	5.62	3.32	2.05	5.86	4.12	1.73
Total variance (%)	37.44	22.12	13.67	39.05	27.45	11.51
Cumulative eigenvalue	5.62	8.94	10.99	5.86	9.98	11.71
Cumulative percentage	37.44	59.56	73.23	39.05	66.5	78.01
